# Supplementary material for: Predicting visual function by interpreting a neuronal wiring diagram
Source: Nature. 2024 Oct 2;634(8032):113–23. doi: 10.1038/s41586-024-07953-5 (PMC11446822; doi:10.1038/s41586-024-07953-5)
Supplement: Supplementary file 6 — CRF and ERF predictions for individual TmY4 and TmY9 cells. Analogous to Supplementary Data 3, but for TmY target types. Shown are the top four monosynaptic pathways, the strongest pathway passing through each of the top ten intermediary types (ranking from Extended Data Fig. 7), and the trisynaptic pathway Tm1–TmY–Dm3–TmY (see the section entitled Prediction of spatial normalization). [file 41586_2024_7953_MOESM6_ESM.zip › DataS4/TmY9qΓèÑ/720575940630418257.pdf]

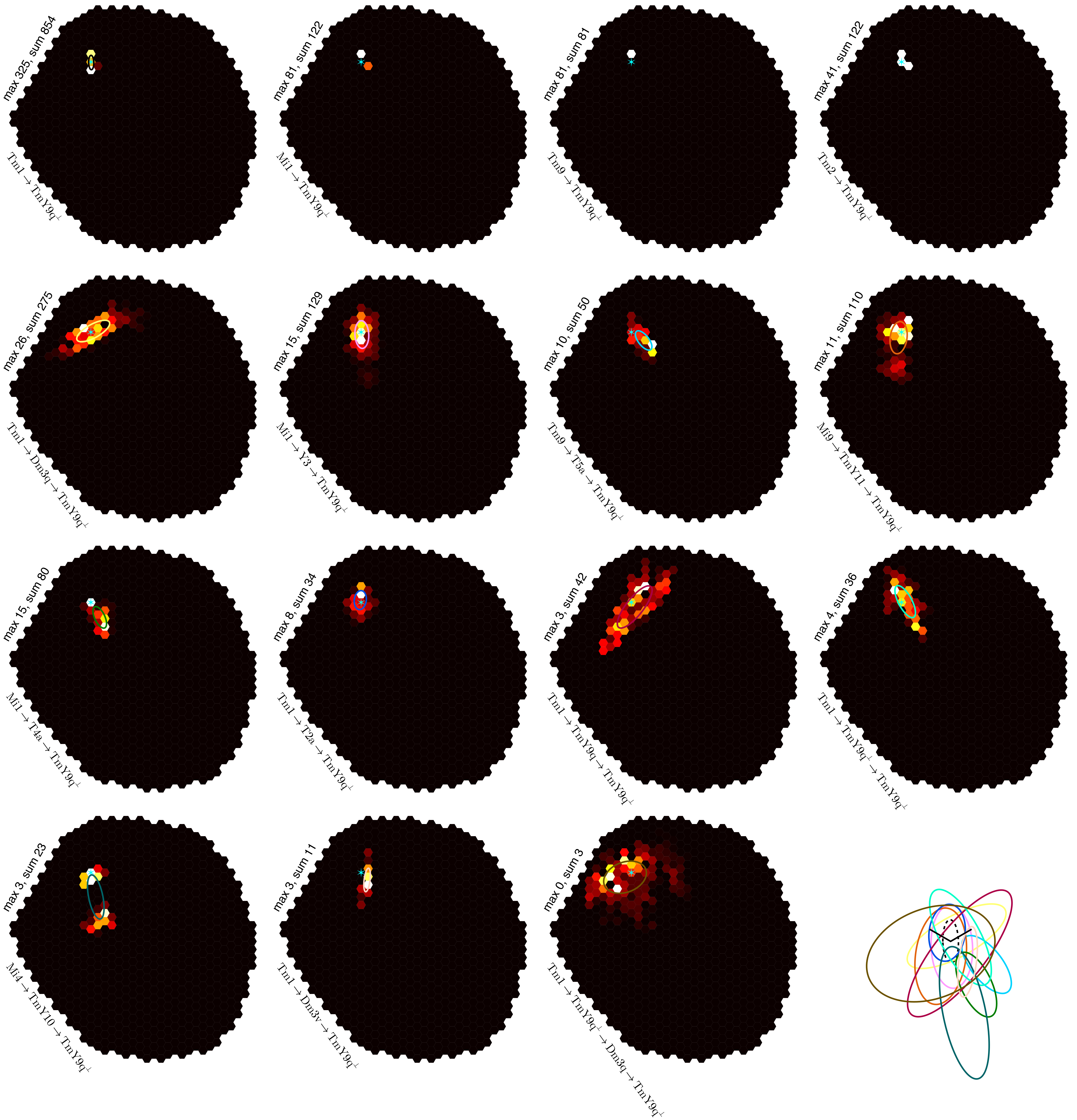

max 325, sum 854
Tm1 → TmY9q⊥
max 81, sum 122
Mi1 → TmY9q⊥
max 81, sum 81
Tm9 → TmY9q⊥
max 41, sum 122
Tm2 → TmY9q⊥
max 26, sum 275
Tm1 → Dm3q → TmY9q⊥
max 15, sum 129
Mi1 → Y3 → TmY9q⊥
max 10, sum 50
Tm9 → T5a → TmY9q⊥
max 11, sum 110
Mi9 → TmY11 → TmY9q⊥
max 15, sum 80
Mi1 → T4a → TmY9q⊥
max 8, sum 34
Tm1 → T2a → TmY9q⊥
max 3, sum 42
Tm1 → TmY9q → TmY9q⊥
max 4, sum 36
Tm1 → TmY9q⊥ → TmY9q⊥
max 3, sum 23
Mi4 → TmY10 → TmY9q⊥
max 3, sum 11
Tm1 → Dm3v → TmY9q⊥
max 0, sum 3
Tm1 → TmY9q⊥ → Dm3q → TmY9q⊥
